# TPST2-mediated receptor tyrosine sulfation enhances leukocidin cytotoxicity and *S. aureus* infection

**DOI:** 10.3389/fimmu.2023.1242330

**Published:** 2023-08-21

**Authors:** Jie He, Xianggui Yang, Kai Yang, Honglin Xu, Cheng Chen, Junxiong Wang, Jun Zeng

**Affiliations:** ^1^ Division of Pulmonary and Critical Care Medicine, Clinical Medical College and The First Affiliated Hospital of Chengdu Medical College, Chengdu, China; ^2^ Department of Laboratory Medicine, Clinical Medical College and The First Affiliated Hospital of Chengdu Medical College, Chengdu, China; ^3^ Chengdu Medical College, Chengdu, China

**Keywords:** sepsis, macrophage, TPST2, tyrosine sulfation, leukocidin

## Abstract

**Background:**

An essential fact underlying the severity of *Staphylococcus aureus* (*S. aureus*) infection is the bicomponent leukocidins released by the pathogen to target and lyse host phagocytes through specific binding cell membrane receptors. However, little is known about the impact of post-transcriptional modification of receptors on the leukocidin binding.

**Method:**

In this study, we used small interfering RNA library (Horizon/Dharmacon) to screen potential genes that affect leukocidin binding on receptors. The cell permeability was investigated through flow cytometry measuring the internalization of 4′,6-diamidino-2-phenylindole. Expression of C5a anaphylatoxin chemotactic receptor 1 (C5aR1), sulfated C5aR1 in, and binding of 6x-His–tagged Hemolysin C (HlgC) and Panton-Valentine leukocidin (PVL) slow-component to THP-1 cell lines was detected and analyzed via flow cytometry. Bacterial burden and Survival analysis experiment was conducted in WT and myeloid TPST-cko C57BL/6N mice.

**Results:**

After short hairpin RNA (shRNA) knockdown of TPST2 gene in THP-1, HL-60, and RAW264.7, the cytotoxicity of HlgAB, HlgCB, and Panton–Valentine leukocidin on THP-1 or HL-60 cells was decreased significantly, and the cytotoxicity of HlgAB on RAW264.7 cells was also decreased significantly. Knockdown of TPST2 did not affect the C5aR1 expression but downregulated cell surface C5aR1 tyrosine sulfation on THP-1. In addition, we found that the binding of HlgC and LukS-PV on cell surface receptor C5aR1 was impaired in C5aR1^+^TPST2^−^ and C5aR1^−^TPST2^−^ cells. Phagocyte knockout of TPST2 protects mice from *S. aureus* infection and improves the survival of mice infected with *S. aureus*.

**Conclusion:**

These results indicate that phagocyte TPST2 mediates the bicomponent leukocidin cytotoxicity by promoting cell membrane receptor sulfation modification that facilitates its binding to leukocidin S component.

## Introduction

*Staphylococcus aureus* (*S. aureus*) is a common pathogen that could cause a wide spectrum of diseases, ranging from mild skin and soft tissue infections to severe diseases, such as pneumonia, bacteremia, and sepsis (1). Antibiotic treatment of *S. aureus* infection is often defeated, and this may partially due to the combination of the production of a vast array of virulence factors and antibiotic resistance. In particular, the pathogenesis of this bacterium is exacerbated by the array of virulence factors that promote immune evasion through the blockade and killing of phagocytes, manipulation of T-lymphocyte and B-lymphocyte responses, and inhibition of complement activation (2). In view of the multiple functions of virulence factors produced by *S. aureus*, it is expected that numerous attempts on developing effective immunotherapies or vaccines have been disappointed when therapeutics have been approached on the basis single target (3). It will be essential to develop novel therapies that simultaneously combat multifaceted pathogenesis of *S. aureus* (4).

Bicomponent pore-forming toxins (PFTs) are the crucial leukocidins secreted by *S. aureus*, including theγ-hemolysin (HlgAB and HlgCB), Panton–Valentine leukocidin (PVL), leukocidin E/D (LukED), and leukocidin A/B (LukAB; also known as LukGH). The two individual components are defined as S (slow migrating) or F (fast migrating) subunits based on column chromatography. These PFTs are capable to manipulate the innate and adaptive immune responses by targeting immune cell receptors (5). Upon binding, these inactive monomeric PFTs oligomerize to form an active octameric pore that spans the phospholipid bilayer of target host cells, causing osmotic imbalance and cell lysis (5, 6). The majority of the proteinaceous receptors that are targeted by bicomponent leukocidins have been identified as class A rhodopsin–like G protein–coupled receptors (GPCRs), including CXC chemokine receptor 1 (CXCR1), CXC chemokine receptor 2 (CXCR2), C5a anaphylatoxin chemotactic receptor 1 (C5aR1), C5a anaphylatoxin chemotactic receptor 2 (C5aR2), CC-chemokine receptor 2 (CCR2), CC-chemokine receptor 5 (CCR5), and Duffy antigen receptor for chemokine (DARC) receptor (3). The CXCR1 and CXCR2 have been identified as targets of HlgAB (7). The CCR5, as well as CXCR1 and CXCR2, was targeted by LukED (8, 9). The DARC was targeted by HlgAB and LukED (10). Moreover, the C5aR1 and C5aR2 have been identified as targets for HlgCB and PVL (7, 11). Although these receptors interacting with bicomponent leukocodins have been identified, little is known about the characteristics of the interaction. In addition, molecular mechanisms underlying the leukocidin-receptor interactions are not entirely known.

Sulfation modification on tyrosines contributes negative charge in the NH2-terminal region of CCR5 and facilitates HIV-1 entry. Inhibition of tyrosine sulfation substantially decreases the affinity of Human Immunodeficiency Virus 1 (HIV-1) gp120/CD4 complexes for CCR5 (12, 13). Tyrosine sulfation plays a role in chemokine binding, and the high density of tyrosines in the N-terminal region of many chemokine receptors suggests that sulfation modifications may commonly contribute to ligand binding to receptors (14).

To determine whether the post-translational tyrosine sulfation involved in the binding of leukocidins to their respective GPCR host counterparts, we applied a small interfering RNA (siRNA) library screen to identify host factors involved in HlgAB, HlgCB, and PVL cytotoxicity. We identify tyrosine sulfation pathways that enhance receptor-mediated susceptibility of human phagocytes to leukocidins. Sulfation modification of phagocyte receptors serves as an enhancer for their interaction and promotes *S. aureus* infection.

## Experimental procedures

### Recombinant protein production and purification

Clinically isolated *S. aureus* strains that were used to amplify coding sequences of leukocidins are listed in **Supplementary Table 1**. Polyhistidine-tagged HlgA, HlgB, HlgC, LukS-PV, and LukF-PV in this study were cloned and expressed by pET303-6x-His plasmid, as described elsewhere (15). Briefly, the coding sequences without encoding the signal peptide were cloned and transformed into *E. coli* BL21-pLyss. To obtain the high expression of polyhistidine-tagged protein, isopropyl-β-d-thiogalactoside (IPTG) was added during exponential growth of transformed *E. coli*. Target proteins were purified from supernatants of cell lysates using a HisPur™ Ni-NTA kit (Thermo Scientific, no. 90099). The *E. coli* endotoxin Lipopolysaccharide (LPS) was removed using a Pierce™ kit (Thermo Scientific, no. 88276).

### Bacterial strain culture and typing

Isolated *S. aureus* strains used during this study are shown in **Supplementary Table 1**. In-frame deletion of gamma toxin genes (ΔhlgABC) from the clinically isolated *S. aureus* CI6 strain were constructed by the use of a pMAD plasmid, as described previously (16). The method to produce implementation of HlgACB mutants is described in a previous study (7). Specific primers that were designed are shown in **Supplementary Table 2**. Clinical *S. aureus* strains CI6, isolated from bronchoalveolar lavage fluids, were originated from patients admitted to the Respiratory Intensive Care Unit of The First Affiliated Hospital of Chengdu Medical College, China. The vitek 2 automatic microbiological identification analyzer and 16s ribosomal RNA (rRNA) sequencing were applied for microbial identification, and disk diffusion was used to perform antimicrobial susceptibility. All strains were cultured in trypticase soy broth or brain heart infusion broth, and overnight supernatants of *S. aureus* culture were filter-sterilized. For animal experiments, mid-exponential subcultures of experimental strain were washed adequately in Phosphate Buffer Saline (PBS). All isolated *S. aureus* strains were typed using the multilocus sequence typing method (17). PCRs were performed on *S. aureus* genomic DNA for amplifying leukocidin S component, and specific primers that were designed are shown in **Supplementary Table 3**.

### *S. aureus* RNA extraction and cDNA synthesis

RNA was extracted from *S. aureus* samples by a TRIzol kit (Thermo scientific) following the manufacturer’s instructions. Overnight cultured bacteria were diluted in fresh medium and continue to culture until early stationary phase (6.5 h; OD660 ± 1.5). Bacteria were centrifuged at 12,000 rpm for 30 s. Centrifugal precipitation were kept in −80°C to stop cell metabolism and resuspended in TRIzol buffer. Contaminating DNA was eliminated by consecutive mix of samples with a DNase I, RNase-free kit (Thermo Scientific). Total RNA quantification was detected by an ultraviolet spectrophotometer Evolution 350 (Thermo Scientific), and quality was verified again on agarose gel eletrophoresis. Contamination with DNA was excluded by a PCR against chromosomal DNA. Isolated equal RNA was used for synthesis of complementary DNA (cDNA) by the cDNA Synthesis Kit for RT-qPCR (Thermo Scientific).

### Hematoxylin and eosin staining and immunoblotting

Lungs were excised, and the right lungs were fixed in 4% formaldehyde for 24 h and then embedded in paraffin. The paraffin-embedded lung tissues were cut and stained by hematoxylin and eosin. Immunoblotting assay was performed, as described previously (18).

### Quantification of phagocyte receptor expression levels and binding assays

Phagocyte receptor expression levels were quantified, as described elsewhere (7). Briefly, THP-1 cells were suspended in a total of 5 mL at 5 × 10^6^/mL with mouse anti-human monoclonal antibodies (mAbs; 10 µg/mL) for C5aR1 (sulfation-independent clone S5/1, AbD Serotec, Kidlington, UK) and mouse anti-human mAbs for C5aR1 (sulfation-dependent clone 347214, R&D Systems, Abingdon, UK), followed by the fluorescein isothiocyanate–conjugated goat anti-mouse secondary antibody (1:50, LifeSpan BioSciences, Seattle, WA, USA). Samples were subsequently analyzed by flow cytometry. To measure the leukocidin S-component binding on THP-1 cells, they were incubated with polyhistidine-tagged HlgC or LukS-PV (20 µg/mL) for 25–30 min at room temperature followed by mouse anti–his-fluorescein isothiocyanate (FITC) antibody (1:30, LifeSpan BioSciences, Seattle, WA, USA). Cells were subsequently analyzed by flow cytometry.

### Cell lines and transfections

THP-1 cells (a human monocytic leukemia cell line) stably transfected with C5aR1 or an empty expression vector. Human C5aR1, residing on a single exon, was cloned into a pcDNA3.1 vector (Invitrogen), as described previously (11). Amplification reactions were performed on Applied Biosystems of the previous cloned humanized cells using PrimeSTAR^®^ Max DNA Polymerase (TaKaRa). Primers and restriction sites used during the study are listed in **Supplementary Table 4**. For stable expression, the target plasmids were transfected into THP-1 cells using Lipofectamine 3000 (Invitrogen). Cells 24 h after transfection were cultured under antibiotic selective pressure using puromycin (1 µg/mL) in Dulbecco's modified eagle medium (DMEM) with 10% fetal calf serum. After 2 to 3 weeks, cells were subcloned and tested for stable expression of C5aR1 using flow cytometry. The expression of human C5aR1 on transfected THP-1 cells was defined using mouse anti-human mAbs (10 µg/mL) for C5aR1, followed by the Allophycocyanin (APC)-labeled goat anti-mouse secondary antibody (1:50, DAKO). Receptor expression was subsequently analyzed by flow cytometry. The human promyelocytic leukemia cell line HL-60 was cultured in Roswell Park Memorial Institute (RPMI) 1640 medium (Gibco, Grand Island, NY, USA) with 10% Foetal Bovine Serum (FBS) (Gibco, Grand Island, NY, USA) and 2 mM L-glutamine (HyClone, Logan, UT, USA) in a 37°C incubator with a humidified 5% CO_2_ atmosphere. The murine macrophage cells RAW264.7 were stored in our laboratory. The cells were cultured in DMEM with 10% FBS, streptomycin (100 µg/mL), and penicillin (100 U/mL) and at 37°C with 5% CO_2_. For experiments, the above cells (seeded at 4 × 10^5^ cells/mL) were challenged by LPS (100 ng/mL) for 8 h, as described previously (19).

### Myeloid TPST2^flox+/−^ mice construction and *in vivo* experiments

We used Clustered regularly interspaced short palindromic repeats--associated protein 9 (CRISPR-cas9) technique to construct TPST2^flox+/−^ and Cre mice. The guide RNA (gRNA) (gRNA1 reverse: GTCCTCTGTGCCCGACGTCAGGG; gRNA2 forward: ACATAAAAGTCACCCACGTGTGG) to mouse TPST2 gene, the donor vector containing loxP sites, and Cas9 mRNA were co-injected into fertilized mouse eggs to generate targeted conditional knockout (cko) offspring. F0 founder animals were identified by PCR (5′ arm forward primer (F1): 5′-CTGTGTCAGTCACCAGCCATAG-3′; 3′ loxP reverse primer (R1): 5′-GTGGATTCGGACCAGTCTGA-3′) followed by sequence analysis, which were bred to wild-type (WT) mice to test germline transmission and F1 animal generation. Heterozygous TPST2^flox+/−^ targeted mice were inter-crossed to generate homozygous targeted TPST2^flox+/+^ mice; then, homozygous TPST2^flox+/+^ targeted mouse was breed with a myeloid-specific Lyz-Cre delete mouse to generate mice that are hemizygous for a targeted allele and a hemizygous for the Cre transgene. Hemizygous Cre^+^ mice were breed with homozygous mice; approximately 25% of the progeny from this mating will be homozygous for the targeted allele and hemizygous for the Cre transgene. The pups can be screened by the PCR (F: 5′-CTTGAGTGCCAACTAAGTCCCAAG-3′; R: 5′-AATGATGGGATGAGTGCCTGAATG-3′; homozygous: one band with 308 bp; heterozygous: two bands with 308 and 240 bp; WT: one band with 240 bp) and immunoblot assay. The tissue-specific gene deletion can be confirmed by adding one additional primers (F: 5′-CTTGAGTGCCAACTAAGTCCCAAG-3′; R: 5′-AATGATGGGATGAGTGCCTGAATG-3′; wild type: one band with 240 bp; no Cre activity: one band with 308 bp) and primer (F: 5′-CTTGAGTGCCAACTAAGTCCCAAG-3′, R: 5′-TAACTTCAGTGTGCCCGGTTAG-3′; with Cre activity: one band with 293 bp) to the PCR assay.

Eight- to 12-week-old age- and gender-matched mice were intraperitoneally injected with 1 mL of PBS containing 5 × 10^8^ colony-forming units (CFU) of bacteria or intravenously injected with 0.5 mL of PBS containing 1 × 10^8^ CFU of bacteria. At 24 h after infection, peripheral blood was obtained, and mice were killed to obtain the lung and spleen tissues. Peripheral blood samples were plated to evaluate the bacterial burden. The lung and spleen tissues (1 g) were fully milled with a tissue homogenizer and then suspended in 1 mL of PBS and plated to evaluate the bacterial burden. In the sepsis mice with intraperitoneally infection, mortality was compared between different groups.

### Cell permeability assays

For permeability assays, THP-1 cell line (1 × 10^7^ cells/mL in RPMI with 10% FBS) was exposed to recombinant toxins and incubated for 30 min at 37°C. The cell lines were subsequently analyzed by flow cytometry, and pore formation was determined by intracellular staining with 4′0,6-diamidino-2- phenylindole (DAPI; 2.5 µg/mL) (20). As HlgAB, HlgCB, and PVL are bicomponent toxins, equimolar concentrations of polyhistidine-tagged S-component and F-component proteins were used. Pore formation ability was defined as the collective DAPI internalization at different concentrations during 30 min. A lactate dehydrogenase (LDH) release assay kit (Merck, Darmstadt, Germany), operating according to the manufacturer’s instruction, was also used to evaluate the cell cytotoxicity by toxins.

### Graphical and statistical analyses

Flow cytometry analyses were performed by FlowJo 10. Data analyses were performed by Graphpad Prism 9.0. Statistical significance was calculated using ANOVA and Student’s t-tests with standard error of mean (SEM) where appropriate. Determination of half-maximal effective lytic concentrations of leukocidins was used in nonlinear regression analyses. All comparisons survival analyses were performed using Gehan–Breslow–Wilcoxon test, unless otherwise indicated. P-values are stated as follows: *P < 0.05, **P < 0.01, ***P < 0.001, and ****P < 0.0001.

## Result

### Phagocyte TPST2 mediates the bicomponent leukocidin cytotoxicity

**Figure 1:**
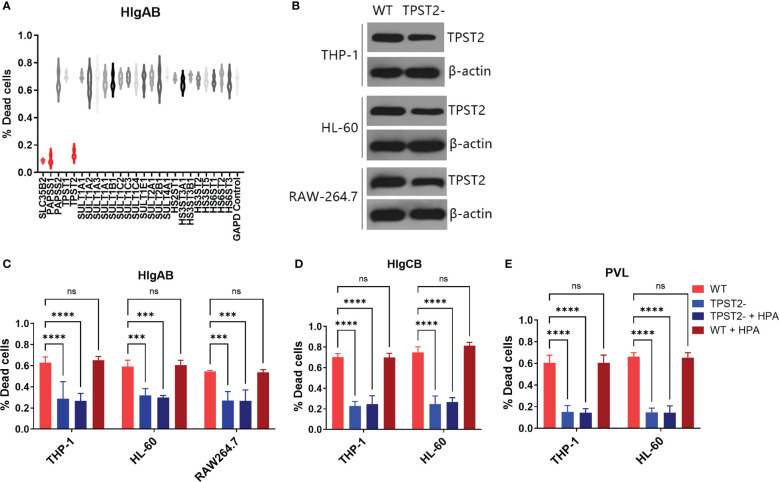
TPST2 promotes the cytolytic activity of bicomponent leukocidins. **(A)** Sulfation-related siRNA library screened target genes regulating HlgAB cytotoxicity tested by LDH release assay. **(B)** TPST2 was knockdown by shRNA lentivirus in THP-1, HL-60, and RAW264.7 cells; immunoblotting was used to detect TPST2 expression. **(C–E)** LDH release assay was used to evaluate cytotoxicity of HlgAB, HlgCB, and PVL in the indicated cell lines transduced with control shRNA, TPST2, shRNA treated with or without the HPA. **(C–E)** Results are the means ± SEM, with n = 3 different donors. “-”indicates shRNA knockdown. ***P < 0.001 and ****P < 0.0001; ns indicates no significant.

To identify host protein modifications that are implicated in leukocidin S-component binding, we first screened siRNA library (Horizon/Dharmacon) containing 25 customized sulfation-related genes with THP-1 cells, which are HlgAB-sensitive macrophage-like cells differentiated from the human leukemia cell line (21). After solute carrier family 35 member B2 (SLC35B2), 3'-phosphoadenosine 5'-phosphosulfate synthase 1 (PAPSS1), or tyrosylprotein sulfotransferase 2 (TPST2) siRNA interference, THP-1 cells became highly resistant to purified HlgAB (HlgA + HlgB), as measured by an LDH release assay *in vitro* (**Figure 1A**), which suggested that PAPSS1/SCL35B2/TPST2 tyrosine sulfation signal pathway might regulate the cytolytic activity of leukocidins. After shRNA knockdown of TPST2 gene in THP-1, HL-60, and RAW264.7, the cytotoxicity of HlgAB, HlgCB, and PVL on THP-1 or HL-60 cells was decreased significantly, and the cytotoxicity of HlgAB on RAW264.7 cells was also decreased significantly (**Figures 1B–E**). Meanwhile, we found that heparinase II (HPA) did not affect the cytolytic activity of HlgAB, HlgCB, and PVL on those cells, and the effect of heparin (HS) on leukocidin cytotoxicity was excluded (**Figures 1B–E**).

### Tyrosine sulfation of membrane receptors facilitates its binding to leukocidins S component

**Figure 2:**
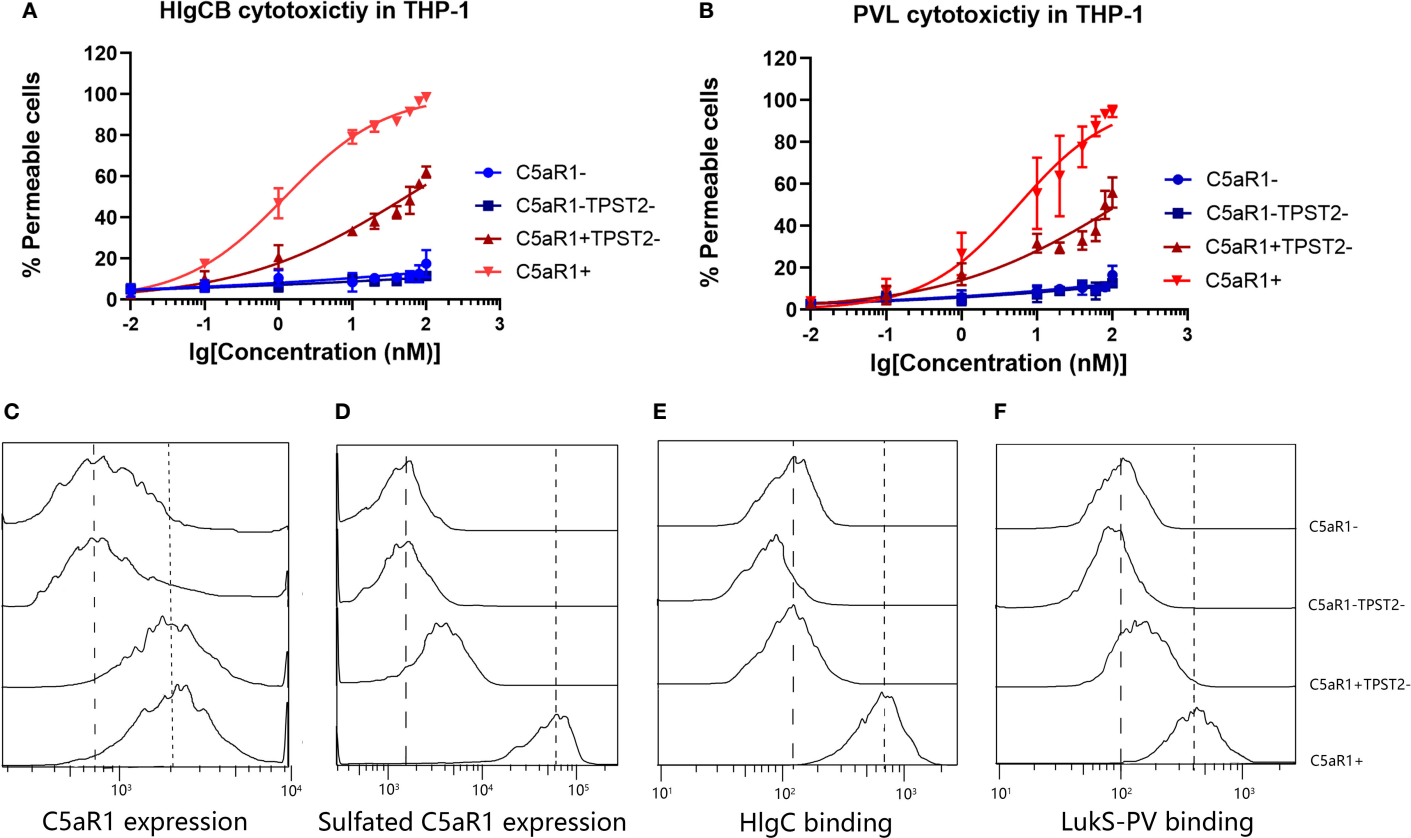
TPST2 promotes the tyrosine sulfation of membrane receptors and its binding to leukocidins S component. **(A, B)** Susceptibility of THP-1 cells transduced with shRNA for C5aR1 (C5aR1^−^), C5aR1 and TPST2 (C5aR1^−^TPST2^−^), TPST2 (C5aR1^+^TPST2^−^), and non-targeting control shRNA (C5aR1^+^) to HlgCB and PVL. As a readout for cell permeability, flow cytometry was used to measure internalization of DAPI at 20 min after leukocidin treatment at 20 nM HlgCB or 10 nM PVL. **(C)** The expression of C5aR1 and sulfated C5aR1 and the binding of 6x-His-tagged HlgC and LukS-PV to THP-1 cell lines transduced with shRNA for C5aR1 (C5aR1−), C5aR1 and TPST2 (C5aR1^−^TPST2^−^), TPST2 (C5aR1^+^TPST2^−^), and non-targeting control shRNA (C5aR1^+^). **(C–F)** Anti-C5aR1 (Invitrogen, clone 20/70) and anti-C5aR1 (R&D Systems, clone 347214) antibodies were used to assess the expression of C5aR1 and sulfated C5aR1 separately. Anti–6x-His-FITC antibodies were directly used to detect the binding of HlgC or LukS-PV. Fluorescence was detected and analyzed *via* flow cytometry. Dotted line, expression on and binding to C5aR1^+^ THP-1 cells; dashed line, expression on and binding to C5aR1^−^ THP-1 cells. Histograms depict the representative examples of two independently repeated experiments.

C5aR1^−^, C5aR1^−^TPST2^−^, C5aR1^+^TPST2^−^, and C5aR1^+^ cells were challenged with HlgCB and PVL. Consistent with the screening results, C5aR1^−^, C5aR1^−^TPST2^−^, and C5aR1^+^TPST2^−^ cells showed resistance to pore formation induced by both HlgCB and PVL (**Figures 2A, B**). To investigate the mechanism of TPST2 regulating HlgCB and PVL cytotoxicity, single-knockdown cells were generated in THP-1 cells. Single-knockdown cells were incubated with specific antibodies to detect the expression of specific targets and analyzed using flow cytometry (20). Knockdown of TPST2 did not affect the C5aR1 expression, as determined using a sulfation-independent anti-C5aR1 antibody (Invitrogen, clone 20/70; **Figure 2C**) but downregulated cell surface C5aR1 tyrosine sulfation, as determined using an anti-sulfotyrosine antibody (R&D Systems, clone 347214; **Figure 2D**) (22). Lack of tyrosine sulfation did not affect the overall levels of C5aR1 expression. These results identify tyrosine sulfation, as a conservative post-translational modification (PTM) mediates the interaction between leukocidins and host cells.

Previous study reported that the binding of the S-component LukS-PV to a synthetic N-terminal C5aR1-peptide was regulated by sulfation of two peptide-tyrosine residues (11). We hypothesized that TPST2 knockdown in THP-1 cells reduces C5aR1 sulfation and subsequent binding of the S components HlgC and LukS-PV. Finally, we found that the binding of HlgC and LukS-PV on cell surface receptor C5aR1 was impaired in C5aR1^+^TPST2^−^ and C5aR1^−^TPST2^−^ cells as expected (**Figures 2E, F**). Thus, these results show that C5aR1 post-translational sulfation modification mediates the host cell susceptibility to both HlgCB and PVL.

### Phagocyte knockout of TPST2 protects mice from *S. aureus* infection

**Figure 3:**
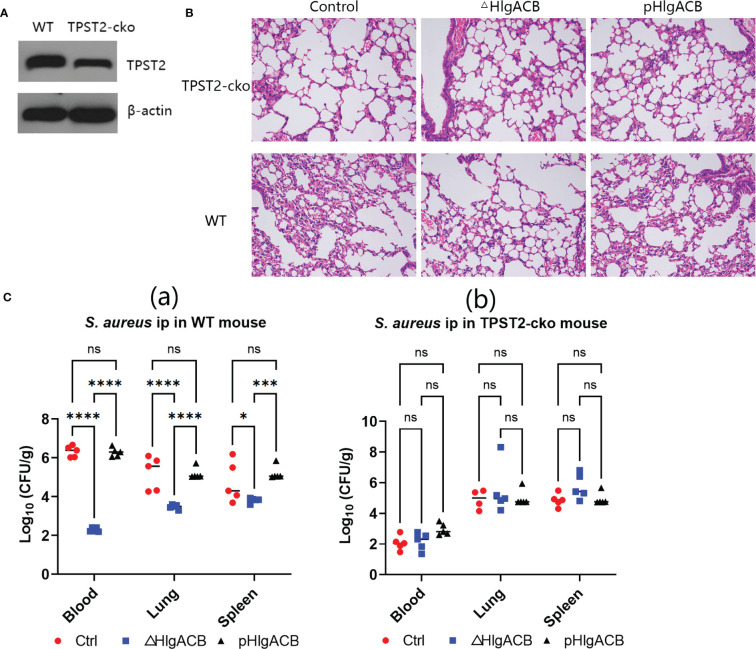
Phagocyte TPST2 knockout lowers *S. aureus* pathogenicity. **(A)** Immunoblotting was used to detect the TPST2 expression level of blood in WT and myeloid TPST-cko C57BL/6N mice. **(B)** WT and myeloid TPST-cko mice were infected intravenously with 1 × 10^8^ colony-forming units (CFU) control, △HlgACB, and pHlgACB *S. aureus* for 24 h; the resected lungs were stained with hematoxylin and eosin (HE). **(C)** Bacterial loads in the blood, lungs, and spleen after infection intraperitonealy with 5 × 10^8^ CFU control, △HlgACB, and pHlgACB *S. aureus* in WT and myeloid TPST-cko mice for 24 h. **(C)** Results are the means ± SEM, with n = 3 different donors. CFU from individual mice are shown means ± SEM, with n = 5 mice per group. Statistical analysis of bacterial burden was performed using ANOVA with Bonferroni correction for multiple comparisons, *P < 0.05, ***P < 0.001, and ****P < 0.0001; ns indicates no significant.

To identify the role of host TPST2 in the immunopathological change of mice infected with *S. aureus in vivo*, the WT and myeloid cell TPST2-cko mice were infected with equal amounts of control (clinical isolated *S. aureus* strains express bicomponent leukocidins HlgA, HlgB, and HlgC), HlgACB-knockout (△HlgACB), and HlgACB-complemented (pHlgACB) *S. aureus* strains intraperitoneally. The lung injury was examined in experimental mice at 24 h after infection; we found that myeloid TPST2 knockout could attenuate the inflammation response of acute lung injury in mice infected by different types of *S. aureus* and that the pathogenicity of △HlgACB *S. aureus* infection was significantly lowered in experimental mice compared with that of control and complemented pHlgACB *S. aureus* (**Figures 3A, B**). In addition, the bacterial load in the blood, lung, and spleen was further detected. In WT mice, HlgACB knockout in *S. aureus* significantly lowered the bacterial load in the blood and lung compared with that in control and pHlgACB *S. aureus*, and the bacterial load was slightly reduced in the spleen infected by △HlgACB *S. aureus* compared with that by others may be because of its immune organ property (**Figure 3Ca**). There exists no difference for bacterial load in the blood, lung, and spleen in TPST2-cko mice infected by different types of *S. aureus* (**Figure 3Cb**).

### Phagocyte knockout of TPST2 improves the survival of mice infected with *S. aureus*

**Figure 4:**
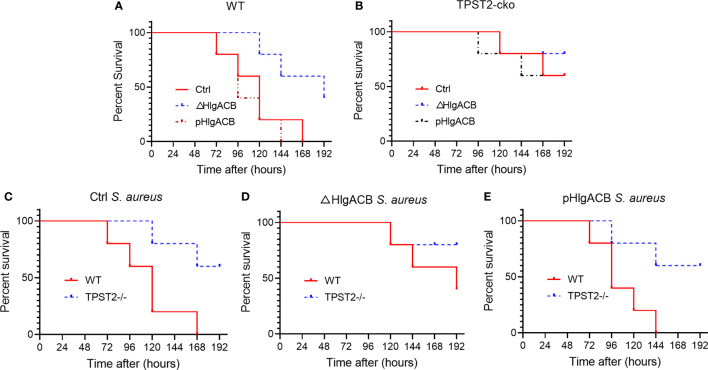
Phagocyte TPST2 knockout improves survival rates of *S. aureus*–infected mice. **(A)** Survival analysis of WT mice injected intraperitoneally with 5 × 10^8^ CFU of control, △HlgACB, and pHlgACB *S. aureus* strains within 192 h. n = 10 mice per group. **(B)** Survival analysis of myeloid TPST2-cko mice injected intraperitoneally with 5 × 10^8^ CFU of control, △HlgACB, and pHlgACB *S. aureus* strains within 192 h. n = 8 to 10 mice per group. **(C)** Survival of WT and myeloid TPST2-cko mice infected with 5 × 10^8^ CFU of control *S. aureus* strains within 192 h. n = 8 to 10 mice per group. **(D)** Survival of WT and TPST2-cko mice infected with 5 × 10^8^ CFU of △HlgACB *S. aureus* strains within 192 h. n = 10 mice per group. **(E)** Survival of WT and myeloid TPST2-cko mice infected with 5 × 10^8^ CFU of pHlgACB *S. aureus* strains within 192 h. n = 8 to 10 mice per group. Statistical analysis by log-rank (Mantel–Cox test).

Having identified that TPST2 promotes the infection and pathogenicity of *S. aureus*, next, we further investigated whether the TPST2 could accelerate the death of mice infected by *S. aureus*. For these *in vivo* experiments, we used control, △HlgACB, and pHlgACB *S. aureus* strains to intraperitoneally infected WT and myeloid TPST2-cko mice and then recorded and analyzed the overall survival of experimental mice. We found that knockout of HlgACB toxin could improve survival of WT mice infected by *S. aureus* compared with that by control and pHlgACB *S. aureus* strains (**Figure 4A**). However, once we knocked out the TPST2 gene in myeloid cells, the survival rates of knockout mice infected by different strains were raised, and there exists no distinct difference (**Figure 4B**). Furthermore, we found that the survival rates of myeloid TPST2-cko mice infected by control, △HlgACB, and pHlgACB *S. aureus* strains were significant elevated compared with that of WT mice, which suggests that TPST2 knockout protect mice from infection and lethality by *S. aureus* (**Figures 4C–E**).

## Discussion

Several studies had demonstrated that toxin-triggered macrophage necroptosis predominantly contributed to lung damage during *S. aureus*–induced pneumonia (21, 23). Giving the key role of *S. aureus* PFTs to cause pathogenesis, these toxins have attracted wide interests as potential therapeutic targets (24–26). Recently limited evidence has shown that a number of broadly neutrolizing mAbs, H5 (targets Hla, HlgAB, HlgCB, LukED, and PVL) and SA185 (targets HlgAB, LukED, and PVL), are superior compared with antigen-specific mAbs (27). Meanwhile, a number of anti-alpha toxin mAbs are currently under clinical development for preventing pneumonia caused by *S. aureus* (28–30). Considering a strong precedent for failure in single-target mAb approaches (24), the value of integrating multidisciplinary therapeutics becomes more promising when conceiving anti-staphylococcal strategies. Centyrins were known as antibody mimetics, inhibiting *S. aureus* infectivity through intercepting the cytolytic activity of bicomponent leukocidins, which have been explored in several studies (26, 31). However, effective therapeutic treatments targeting toxins are still missing; further more studies are needed to elucidate their mechanisms of action.

PTMs endow a protein functional diversity by regulating structure and function and altering localization and interactions. Their dysregulation causes many diseases (32). PTMs of host receptors are important for regulating association of the membrane receptors with corresponding ligands (22, 33, 34). We used a sulfate-associated genes siRNA library to screen for host factors involved in regulating susceptibility toward receptor-mediated leukocidins and identified SCL35B2/PAPSS1/TPST2 pathways (sulfation modification) driving phagocyte receptor–mediated susceptibility to leukocidins.

Interruption of the sulfation pathway leads to a reduction of S-component binding to target receptors on THP-1 cells, supporting the viewpoint that cell membrane receptor sulfation increases susceptibility of phagocyte cell to leukocidins *via* enhancing binding of the S components (11, 22). Sulfation-deficient C5aR1-expressing THP-1 cells have shown a reduced susceptibility to both HlgCB and PVL. This siRNA screening identifies sulfation-modified receptor employment as a primary and common trait for C5aR1-interacting leukocidins. Previous studies have shown that sulfation of CXCR2-expressing cells lacks an apparent influence on HlgAB and LukED cytotoxicity, because of its lack of sulfation modification motifs on CXCR2 N-terminus that mediate TPST2 activity (22). This is probably due to less efficiency of CXCR2 sulfation modification (35). These results indicate that a distinct role for receptor sulfation in leukocidin susceptibility exists. In contrast to the tyrosine sulfation in C5aR1, sialylation is the primary PTM motif enhancing cytotoxicity of CXCR2-targeting leukocidins (22).

The conformational changes of the receptors, especially the extracellular domains, may be involved in the process of hetero-oligomerization, binding of ligands, and pore formation (36). Apart from directly regulating receptor expression levels and facilitating its binding to ligands, tyrosine sulfation also possibly causes conformational changes of the receptors (34). These results identify tyrosine sulfation of receptors as potential targets for new therapies after infection. Editing of specific genes involved in SCL35B2/PAPSS1/TPST2 sulfation pathway resulted in disruption of overall tyrosine sulfation in host cells. To best of our knowledge, no existing compounds interfering the tyrosine sulfation pathway have currently been investigated. As both TPST1 and TPST2 knockout mice will die shortly after birth (37), pharmaceutical interference targeting tyrosine sulfation pathway could be challenging on account of the importance of this pathway in maintaining cell homeostasis.

The tyrosine sulfation displays a wide heterogeneity in respect to cell types (34, 38), whereas the tissue tropism of *S. aureus* infections is poorly understood. Sulfation modification variation in different cell types and organs may possibly be in favor of organ-specific *S. aureus* infections. The mechanisms for the interindividual variability toward serious infections by *S. aureus* are poorly understood (39, 40). Variations in the genes encoding tyrosine sulfation pathways might explain differences in susceptibility of humans to severe infections caused by *S. aureus*. More studies are needed to verify the above hypothesis.

## Data availability statement

The raw data supporting the conclusions of this article will be made available by the authors, without undue reservation.

## Ethics statement

The animal study was approved by the Ethics Committee of the First Affiliated Hospital of Chengdu Medical College. The study was conducted in accordance with the local legislation and institutional requirements.

## Author contributions

JZ designed the experiments. JH, XY, and KY conducted the study. CC and JW conducted the mice experiments. HX collected and analyzed data. ZJ wrote the manuscript. All authors read and approved the final manuscript.
