# Supplementary material for: TPST2-mediated receptor tyrosine sulfation enhances leukocidin cytotoxicity and S. aureus infection
Source: Front Immunol. 2023 Aug 21;14:1242330. doi: 10.3389/fimmu.2023.1242330 (PMC10476081; doi:10.3389/fimmu.2023.1242330)
Supplement: Supplementary file 1 [file Table_1.docx]

**Supplementary tables**

1. **Supplementary Table 1 (Related to Methods).**

*S. aureus* strains used in this study.

| ***S.aureus* strain** | **Toxin expression Characteristics** | **Origin** | **Referrence** |
| --- | --- | --- | --- |
| CI1 | LukS-PV、LukF-PV、HlgA、HlgC、LukE、LukA、LukB | Clinical isolate | This study |
| CI2 | HlgA、HlgB、HlgC、LukA、LukB | Clinical isolate | This study |
| CI3 | LukS-PV、LukF-PV、HlgA、HlgB、HlgC、LukA | Clinical isolate | This study |
| CI4 | LukF-PV、HlgA、HlgB、HlgC | Clinical isolate | This study |
| CI5 | LukS-PV、LukF-PV、HlgA、HlgB、HlgC | Clinical isolate | This study |
| CI6 | HlgA、HlgB、HlgC | Clinical isolate | This study |

1. **Supplementary Table 2 (Related to Methods).**

Primer sequences used in this study to homologously knockout target genes on CI5 strain or to produce expression plasmids for complementation of isogenic *S. aureus* mutants (F: Forward primer; R: Reverse primer). Restriction enzyme recognition sites are underlined.

| **Target** | ***E. coli* strain and plasmid** | **Purpose** | **Enzyme** | **Primers** |
| --- | --- | --- | --- | --- |
| HlgA-up | BL21-pLyss,  pMAD | knockout | BamHI/ EcoRI | F: 5’- CG GGATCC ATGGATACAAATCATACATC -3’  R: 5’- CG GAATTC AGAAATCACTTTCTTTC -3’ |
| HlgB-down | *BL21-pLyss*,  pMAD | knockout | Sma I/ Bgl II | F: 5’- CG CCCGGG CTAGTAAAAC ACGGTCGCC -3’  R: 5’- CG AGATCT CATTTAACACAAGAATGTG -3’ |
| pHlgACB | *DH5a*,  pSK265 | Complementation | Sma I/ Hind III | F: 5 ’- CG AAGCTT ATGATTAAAAATAAAATATTAAC -3’  R: 5’- CG CCCGGG CTATTTATTGTTTTCAGTTTC -3’ |

1. **Supplementary Table 3 (Related to Methods).**

Primer sequences used in this study to produce target recombinant leukocidins based on CI5 strain (F: Forward primer; R: Reverse primer). Restriction enzyme recognition sites are underlined.

| **Toxins** | **ACC.No** | **Enzyme** | **Primers** | **Product** |
| --- | --- | --- | --- | --- |
| LukS-PV | gi2696710 | BsmI/ Xho I | F: 5’- CGGAATGCA T GAATCTAAAGCTGATAACAATAATGAG-3’  R: 5’- CCCTCGAG ATTATGTCCTTTCACTTTAATTTC-3’ | 864bp |
| LukF-PV | gi2696710 | Bsm I/ Xho I | F: 5’- CGGAATGCA T GCTCAACATATCACACCTG-3’  R: 5’- CCCTCGAG GCTCATAGGATTTTTTTCCTTAG-3’ | 903bp |
| HlgA | gi295153 | BsmI/ Xho I | F: 5’- CGGAATGCA T GAAAATAAGATAGAAGATATCGG-3’  R: 5’- CCCTCGAG CTTAGGTGTGATGCTTTTAA-3’ | 840bp |
| HlgB | gi295153 | BsmI/ Xho I | F: 5 ’- CGGAATGCA T GAAGGTAAAATAACACCAGTC-3’  R: 5’- CCCTCGAG TTTATTGTTTTCAGTTTCTTTTGTATC-3’ | 867bp |
| HlgC | gi295153 | BsmI/ Xho I | F: 5’- CGGAATGCA T GCTAACGATACTGAAGAC-3’  R: 5’- CCCTCGAG ATTCTGTCCTTTCACC-3’ | 888bp |

1. **Supplementary Table 4 (Related to Methods).**

Primer sequences used in this study to produce expression plasmids for transient transfections (F: Forward primer; R: Reverse primer). Restriction enzyme recognition sites are underlined.

| **Target** | **Enzyme** | **Primers** | **Product** |
| --- | --- | --- | --- |
| **mTPST2** | EcoRI/ Xba I | F: 5’- CG **GAATTC** ATGCGCCTGTCGGTGCGTAAG -3’  R: 5’- CG **TCTAGA AC** CGAACTTCCTAGGTGTGG -3’ | 1128nt |
| **hTPST2** | EcoRI/ Xba I | F: 5 ’- CG **GAATTC** ATGCGCCTGTCGGTGCGGAG -3’  R: 5’- CG **TCTAGA AC** CGAGCTTCCTAAGTGGGAG -3’ | 1131nt |
| **hC5aR1** | EcoRI/ Xba I | F: 5’- CG **GAATTC** ATGGACTCCTTCAATTATACCAC -3’  R: 5’- CG **TCTAGA AC** CACTGCCTGGGTCTTCTGGGC -3’ | 1081nt |
